# Driving and Multitasking: The Good, the Bad, and the Dangerous

**DOI:** 10.3389/fpsyg.2016.01718

**Published:** 2016-11-08

**Authors:** Menno Nijboer, Jelmer P. Borst, Hedderik van Rijn, Niels A. Taatgen

**Affiliations:** ^1^Department of Artificial Intelligence, University of GroningenGroningen, Netherlands; ^2^Department of Psychology, University of GroningenGroningen, Netherlands

**Keywords:** multitasking, interference, working memory, driving simulation, mind wandering, safety

## Abstract

Previous research has shown that multitasking can have a positive or a negative influence on driving performance. The aim of this study was to determine how the interaction between driving circumstances and cognitive requirements of secondary tasks affect a driver's ability to control a car. We created a driving simulator paradigm where participants had to perform one of two scenarios: one with no traffic in the driver's lane, and one with substantial traffic in both lanes, some of which had to be overtaken. Four different secondary task conditions were combined with these driving scenarios. In both driving scenarios, using a tablet resulted in the worst, most dangerous, performance, while passively listening to the radio or answering questions for a radio quiz led to the best driving performance. Interestingly, driving as a single task did not produce better performance than driving in combination with one of the radio tasks, and even tended to be slightly worse. These results suggest that drivers switch to internally focused secondary tasks when nothing else is available during monotonous or repetitive driving environments. This mind wandering potentially has a stronger interference effect with driving than non-visual secondary tasks.

## Introduction

There is a general belief that driving cannot be combined with any other task without affecting driving performance. Several studies have found evidence for this (Ranney et al., 2000), ranging from phone conversations (Strayer and Johnston, 2001; Treffner and Barrett, 2004) to music listening (Brodsky, 2001). However, recent evidence has indicated that multitasking could also be beneficial for driving when the right circumstances are met (Gershon et al., 2009; Atchley and Chan, 2010; Ünal et al., 2012). In this work we develop some theoretical explanations for both decrease and increase in performance, and test these in an experiment for which we predict to see both effects.

### Multitasking interference in driving

When two tasks require the same perceptual or cognitive resource at the same time, they are said to overlap with regards to that resource. Overlap in resource use between concurrently performed tasks leads to contention for those resources (Pashler, 1994; Wickens, 2002; Salvucci and Taatgen, 2008). In turn, this contention typically leads to reduced task performance (e.g., Just et al., 2008; Borst et al., 2010b; Strayer et al., 2013; Nijboer et al., 2014). For our purposes, we will describe overlap in terms of the resources defined in the threaded cognition theory (Salvucci and Taatgen, 2008), which offers an account that is precise in terms of timing, and that is based on resources defined in the ACT-R cognitive architecture (Anderson, 2007). The resources that are most relevant for driving (and secondary tasks next to driving) are the visual perception, auditive perception, declarative memory, working memory and motor control. Although, driving requires all of these resources to some degree (Herbert, 1963; Anstey et al., 2005), the demands on these resources vary depending on the traffic situation. For example, driving on a quiet road mainly requires visual perception and motor control. Moreover, resources are not always required full-time: it is acceptable to look away from the road for short periods of time. According to threaded cognition, resources are assigned to tasks based on two principles: greediness and politeness. The greediness principle states that a task can be used when it is not used by any other task at a particular moment, but has to wait if the resource is in use. The politeness principle states that a task should release a resource as soon as it is not needed anymore. For example, routine driving does not require the use of working memory. Therefore, a secondary task such as having a conversation that does require working memory does not interfere with driving. However, if the driving situation changes in a way that requires working memory, for example planning how to cross a complex intersection, the conversation task may interfere with driving because it does not relinquish working memory soon enough.

Of all the resources that have been studied with respect to driving interference, perceptual and motor interference have been studied most (visual and auditory perception: Chaparro et al., 2005; Gherri and Eimer, 2011; manipulation of equipment: Brookhuis et al., 1991; Briem and Hedman, 1995; Janssen et al., 2012). Cognitive requirements of secondary tasks turned out to be at least as important. Of all tasks found to interfere with driving, cell-phone use has received most attention due to the high number of traffic-accidents attributed to such devices (Redelmeier and Tibshirani, 1997). In an influential study, Strayer and Johnston (2001) showed that it is primarily what they call the attentional component of holding a conversation that disrupts driving performance, by ruling out explanations related to holding the phone, speaking, or listening. Several studies have shown that holding a complex conversation in particular affects driving performance (McKnight and McKnight, 1993; Briem and Hedman, 1995).

A resource that is pivotal in large disruptions of performance in multitasking is working memory. Note that we use a restricted concept of working memory, the part that Baddeley (2012) calls the *central executive*, and Oberauer (2002) the *focus of attention*. It is therefore closely related to Strayer and Johnston's (2001) attentional component. In the threaded cognition theory, focal working memory can hold a single chunk of information. This chunk can, in turn, point to other sources to create a larger context, but it is the only element that is available for immediate information processing. Any other element needs some retrieval or recovery process to use.

Working memory is used to build up temporary representations that are needed in the near future, for example the gist of a conversation (e.g., van Rij et al., 2010, 2013) or to represent the result of partial computations in arithmetic (Borst et al., 2010a). Secondary tasks in driving experiments that involve working memory (e.g., Alm and Nilsson, 1995) typically lead to decrements in both driving performance and performance on the secondary task. The working-memory load of driving is strongly dependent on the traffic: when the road is empty the driver only has to remember information regarding the current state of the car, which can be easily retrieved from visual and aural queues that are constantly present in the environment. When there is substantial traffic, however, the driver has to keep a detailed mental model of the surrounding vehicles (Gugerty, 1997), as these will not always be visible: they might reside in the blind spot of the car, or be obscured by other vehicles.

Whenever there is a situation in which driving suddenly requires working memory, the driver has to give up the contents of working memory for the secondary task, which can lead to severe disruptions in that task. Therefore, the driver may be reluctant to do so, leading to possible dangerous decisions.

Given that accidents in both real and simulated driving are rare, we need a different measure of driving quality. In Alm and Nilsson's (1995) study, the subjects had to follow a leading car, and had to respond to that car's breaking by breaking. The response time was a measure of driving quality. Gershon et al. (2009) used a number of measures for driving quality: lateral deviation from the middle of the lane, where more deviation is associated with less attentive driving, standard deviation in speed, where more deviation indicates the driver pays less attention, and standard deviation in steering angle, where larger angles are indicative for poorer driving. We will use these indicators, with some refinements, in our own study. We will also look at overtaking behavior, where we will use consistency in overtaking distance as a measure of quality, as well as proper turn-signal use, and, given that subjects sometimes do hit other cars, the frequency with which this occurs.

### Beneficial effects of multitasking in driving

In contrast to the findings presented so far, some studies have shown that driving *improves* when concurrently performing another activity. Gershon et al. (2009) showed that a multiple-choice trivia game improved driving performance under monotonous driving circumstances in terms of lateral deviation, standard deviation in speed and standard deviation in steering angle. Atchley and Chan (2010) had similar results with a verbal word-association task in combination with a monotonous driving task. These findings raise an interesting question: what causes performance to improve when a secondary task is introduced?

Research in other areas has also shown that a secondary task can improve performance on the primary activity. For example, doodling on a piece of paper while performing a memory task has been found to improve recognition accuracy by improving overall concentration (Andrade, 2010; Singh and Kashyap, 2015). Andrade (2010) argues that doodling improves performance because it reduces the chance to engage in daydreaming, also referred to as mind wandering. When mind wandering, the attention is shifted away from the task at hand and instead focuses on task-irrelevant thoughts. This behavior will typically occur when tasks have low processing demands, and are thus experiences as boring or repetitive (Giambra, 1995; Forster and Lavie, 2009). This internal focus results in a decoupling of perception and environment (Cheyne et al., 2009; Smallwood and Schooler, 2015), which can have a negative impact on performance of the main task (He et al., 2011). Killingsworth and Gilbert (2010) estimated that up to 50% of everyday life is spent mind wandering. This is in line with research that has shown that people actively seek out opportunities to multitask (Czerwinski et al., 2004; González and Mark, 2004; Gould et al., 2013). Thus, a boring drive might lead to mind wandering, which has been shown to have a negative impact on visual attention during driving (He et al., 2011).

Therefore, adding a secondary task during a monotonous driving setting could have the same effect as doodling has on a boring memory task: it reduces the chance of other, more interfering tasks—such as mind wandering—to intrude the primary activity. This would imply that a secondary task does not make driving performance itself better, but is the lesser of two evils: The interference of mind wandering has a more substantial effect on the driving task than the secondary task does. Alternatively, complete focus on the driving task might lead to drivers over-regulating their driving behavior: according to the execution-focus theory increased attentional control to highly proceduralized sensory-motor skills can disrupt execution of these skills (Baumeister, 1984; Beilock and Carr, 2001). This is typically observed in sports, but might also lead to a decrease in driving skill under circumstances where the cost of failure is significant. We will further discuss those possible explanations after we report our results.

### Current study

The goal of the current study is to study several combinations of secondary tasks and driving scenarios. Driving scenarios consisted of a no-traffic scenario that mainly uses perceptual and motor resources, and a traffic scenario that requires some advance planning to respond to other cars, and therefore requires some additional working memory investment. Secondary tasks consisted of no secondary task, passive radio listening, a radio quiz, and a tablet-based quiz. These tasks (or no task) have increasing working memory demands, and the table-based quiz also has visual perceptual demands. We expect the combinations where working memory is required most of the time, but is never overtaxed, to lead to the best performance. In the case of the no traffic scenario, we expect the radio quiz to lead to the best performance, because it safeguards against mind wandering, and does not interfere very much with driving. In the traffic condition we expect best performance in the no-secondary task condition, because the task demands prevent people from mind wandering.

The most common measure for driving performance is mean deviation from the middle of the lane, based on the idea that lapses in attention to driving lead to drift within the lane. In this study we will investigate several other measures of driving performance: the variability in lane deviation, and maximum deviation, changes in the wheel angle, and in the condition with other traffic, the accuracy of overtaking other cars. Given that our scenarios have no lead car to follow, we ask participants to maintain a constant speed, and will use their ability to do so as an additional measurement.

## Methods

### Paradigm

We created a paradigm that tested the effect of four different secondary activities on driving performance during two different driving scenarios, referred to as the No-Traffic and Traffic scenarios. Driving scenario was a between-subject variable, while the four different secondary tasks were within-subject variables. For each condition we recorded a number of measurements during driving (lane keeping, speed, secondary-task performance, and steering). Both scenarios used a two-lane highway in a desert environment with all traffic driving in the same direction. The road contained two 5-m wide lanes (cf. the minimum highway lane width in the US is 3.7 m AASHTO, 2011, while the standard highway lane width in the Netherlands is 3.5 m) and had a subtle curvature, approximately 3.5 cm of lateral displacement per meter of road, to ensure that minor changes to the car heading needed to be made on a regular basis. The car that was driven was a 1966 Ford Mustang, with a width of approximately 1.7 m and a length of 4.6 m. In both scenarios participants were instructed to drive 80 km/h (50 mph), and to not exceed this speed.

**Figure 1:**
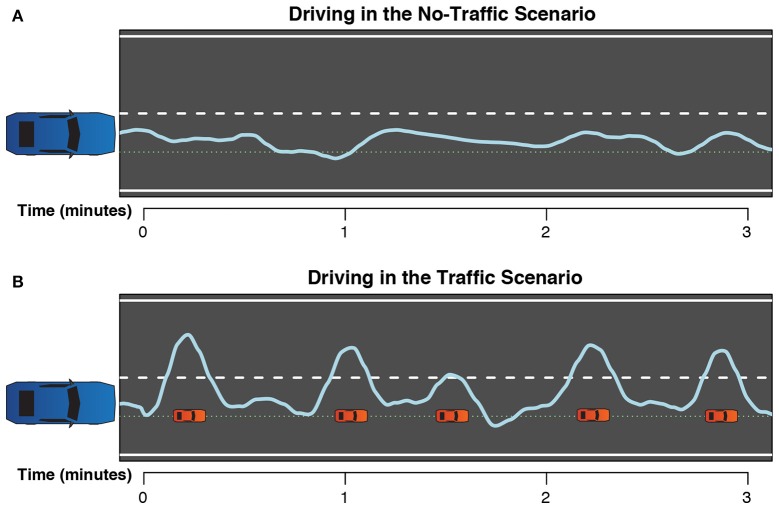
**Two examples of typical driving paths for the two different scenarios**. The blue line is the path taken by the participant. The green dotted line is the center of the right lane, and the white dashed line is the division between the two lanes. Red cars represent slower left-lane traffic that the participant needs to overtake. Driving in the **(A)** No-Traffic and in the **(B)** Traffic scenario.

The No-Traffic scenario was constructed to test the effects of secondary tasks with different resource requirements during situations where the driving itself was easy, and therefore monotonous and boring. In the No-Traffic scenario the highway had no traffic in the right lane: participants were occasionally overtaken by other cars, but did not have to overtake any cars themselves. This is illustrated in Figure 1A. With no relevant traffic to keep track of this scenario only required visual perception and motor control resources.

The Traffic scenario was designed to test how multitasking affects typical highway driving when the road situation requires the maintenance of a mental representation of the traffic situation, and therefore needs working memory as a resource. This was achieved by introducing traffic in both driving lanes. Participants were often overtaken by other cars, and also had to overtake slower cars in the right lane as shown in Figure 1B. The slow right lane traffic was distributed such that at 80 km/h participants would overtake approximately 60 cars during a 30-min block, or 2 cars per min. The distance between right-lane cars varied between 60 and 95 m. The left-lane traffic would overtake the participant at set points, distributed over the span between the previously overtaken right-lane car and the next right-lane car: at approximately 25, 40, 55, and 75% of the total inter-car distance these cars could appear, with a 50% probability for each possibility—so on average participants would be overtaken by two of these cars before having to overtake again themselves.

While most cars in the left lane would overtake the participant at random moments, there were two special types of cars. The first type overtook participants at the time they needed to overtake a slow car in the right lane themselves, forcing them to wait until the faster car had passed. This encouraged participants to keep a mental model of the traffic around them. The second special type of car would drive behind the participant in the left lane at a reasonable distance until the participant overtook a slower car in the right lane. The car would then change lanes to stick behind the slow car. These cars were added to reduce the predictability of left-lane traffic: if all left-lane traffic overtakes the participant, then it would always be optimal to just wait in the right lane until no more traffic can be seen in the rear-view mirror. However, if some cars never pass, this requires a more active role of the participant in anticipating the best time to overtake the leading car. The two special types of cars (who also have a 50% probability to appear, like all other left-lane traffic), together with the random left-lane traffic and randomly spaced slower right-lane cars, created a dynamic highway situation. Because of the chosen setup, the Traffic scenario requires the driver to keep a representation of the immediate environment, and therefore require the use of the working memory resource in addition to visual perception and motor control.

To determine how the working memory, perceptual, and motor requirements of the secondary task interact with driving performance we created four different secondary task conditions. (1) *No Secondary Task Condition (Single)*: In the Single condition there is no secondary task. (2) *Listening Condition*: A radio talk show would play during the entire block. The participants were informed that no information presented in the talk show would need to be recalled later. Therefore, any representation of the content of the show they built during listening (using working memory), could be relinquished without any loss in performance. Furthermore, the aural presentation required a resource (aural) that was not necessary for driving. (3) *Radio-Quiz Condition*: In the Radio-Quiz condition, fragments of a radio talk show, similar to shows in the previous condition, were played split into multiple fragments. A multiple-choice question followed each audio fragment, and participants had to choose between three answers using buttons on the steering wheel. In this condition participants are required to build up a representation of the fragment in working memory. Relinquishing this fragment does have a cost in this condition, because the representation can become fragmented and parts may be forgotten. The motor load is also slightly higher as a button needs to be pressed to respond to the questions. (4) *Tablet-Quiz Condition*: A variation of the Radio Quiz, where all information, both the text of the talk show and the questions, was presented on a tablet in the lower-left corner of the screen, instead of aurally. The working-memory and motor loads are expected to be similar to the Radio Quiz, but the perceptual load is much higher as participants have to shift their gaze from the road to the tablet, as well as the control processes that involve planning voluntary eye-movements (see, for example, Huestegge and Koch, 2009).

### Participants

We recruited 48 native Dutch speakers that were randomly assigned to one of two experimental groups of 24 participants each. The first group drove in the No-Traffic scenario (16 female, *M*_age_ = 24.6, age range: 20–36), while the second group drove in the Traffic scenario (13 female, *M*_age_ = 23.6, age range: 20–32). Both groups contained experienced drivers (No-Traffic: *M*_license_ = 5.0 years, *M*_driven_ = ~65,000 km. Traffic: *M*_license_ = 5.4 years, *M*_driven_ = ~60,000 km). The two groups can be considered comparable, as the Bayes Factors^1^ of the difference between groups for license years and kilometers driven were 0.31 and 0.29 respectively. Participants received a minimum of €20 upon completion, and could earn up to an additional €10 depending on task performance. The average received bonus was €6.40. All participants had normal or corrected-to-normal vision. This study was carried out in accordance with the recommendations of the Ethical Committee Psychology of the University of Groningen with written consent from all subjects. All subjects gave written informed consent in accordance with the Declaration of Helsinki. The protocol was approved by the Ethical Committee Psychology of the University of Groningen.

### Materials and procedure

#### Apparatus

**Figure 2:**
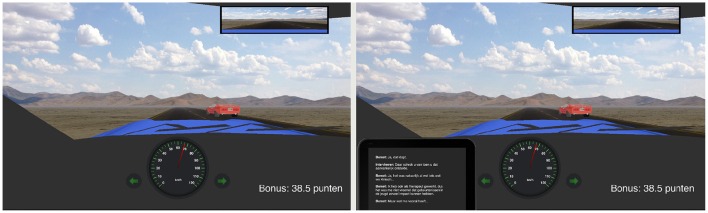
**The simulated driving environment. Left:** The environment during the Single, Passive Listening, and Radio-Quiz conditions. **Right**: The environment during the Tablet-Quiz condition.

The driving scenarios were built and executed in a driving simulator designed and programmed specifically for our paradigm. A steering wheel (Logitech driving force GT) with feet pedals was used to control the car, which had an automatic transmission. The center of the steering wheel contained three buttons on the right side that were used to answer the quiz questions. On the back of the wheel two buttons (one on each side) could be used to activate the left or right turn signal. Participants wore headphones for the auditory stimuli. The simulation was viewed on a 23-inch LCD display at 120 Hz, at a distance of approximately 70 cm from the participant. The simulation environment can be seen in Figure 2. Visible are the hood of the car, the windscreen wipers, a speedometer, turn signal indicators, a rear-view mirror, and the current bonus score. The hood of the car is shown to better judge the road position while driving and to give a sense of size to the information presented in the outside world. Continuous data from the simulation was recorded at 50 Hz. We recorded the car position, pedal pressure, wheel angle, speed, direction indicators, and contact with other cars.

#### Listening stimuli

For the passive radio-listening condition we selected 30-min segments of two episodes of a Dutch popular-science public-radio talk show. The topics of the shows were addiction and music perception. The two episodes were balanced across participants within each scenario, with half of the scenario group listening to the first show, and the other half listening to the second.

#### Radio-quiz stimuli

For the quiz we used two episodes of the same science talk show, but that were different from the ones used in the Listening condition. This time the topics were depression and improving mental health. We generated 30 questions for each episode, with 3 possible answers per question. The Apple OS X text-to-speech function was used to create the audio files. As all stimuli were in Dutch we used the “Xander” voice to ensure intelligibility. Like the listen-only episodes, the presented episode was balanced across participants within each scenario group.

#### Tablet-quiz stimuli

The stimuli for the Tablet Quiz were transcripts of fragments of the episodes and questions used in the radio quiz. The rate of sentence presentation was matched to the length of the original audio fragments, and each presentation of a new sentence was accompanied with a tone sound. The display accommodated a maximum of 10 lines at a time, which covered around 30% of the width of the entire screen. A sentence was on screen for at least 10 s. The radio and tablet quizzes were paired in such a way that participants were not presented the same topic twice.

#### Procedure

The experiment lasted slightly under 2.5 h. Participants started with a 5-min training session to familiarize themselves with the driving task and overtaking other cars. To become accustomed to handling a secondary task while driving, participants performed a second 5-min driving session during which they also carried out the tablet task. The actual experiment consisted of four 30-min blocks, resulting in a drive length of 120 min in total. Therefore each block was slightly longer than the average commute time in the United States (25 min; McKenzie and Rapino, 2011). Each block corresponded to one of the four secondary task conditions, which was performed in the driving scenario that the participant was assigned to. The order of conditions was counter-balanced across participants using a Latin square to avoid order effects. To avoid possible effects of switching between the blocks, the first 5 min of each block were removed from the analysis, leaving 25 min. After each block there was an opportunity for participants to take as long a break as they required before continuing on to the next block.

To motivate participants to perform well, they could increase their financial reward by collecting bonus points. Each bonus point was worth 10 cents, and the starting bonus was 40 points. The maximum bonus was 100 points. Points could be earned during the radio and tablet quiz. Each correct answer was worth 1 point. However, participants could lose points by either driving off the road (−1 point per second off-road), hitting other cars (−2 points per hit), or not signaling properly when changing lanes (−1.5 points per offense).

## Results

Unless mentioned otherwise, all *p*-values of the main effects are from analyses of variance performed on linear mixed-effects models (LME). Accuracy data were modeled using binomial LMEs. The *p*-values of individual comparisons between conditions were computed by performing a Tukey honest significant difference test on each LME. All models were constructed and analyzed in R (3.0.2) with the lme4 package (1.0-5). To assess overall significance of condition, a mixed-effect model without condition was compared to the model that included condition using a χ^2^ test on log likelihood. All error bars in figures depict the upper half of 95% confidence intervals for the mean, corrected for a within-subject design.

Overall, the Tablet-Quiz condition led to the worst driving performance, followed by the Single, no secondary task, condition. The Listening and Radio-Quiz conditions resulted in the best driving performance. This pattern appears in most of the variables that were measured, over both driving scenarios, and therefore appears robust. We will now discuss the separate measures in more detail.

### Secondary tasks

**Figure 3:**
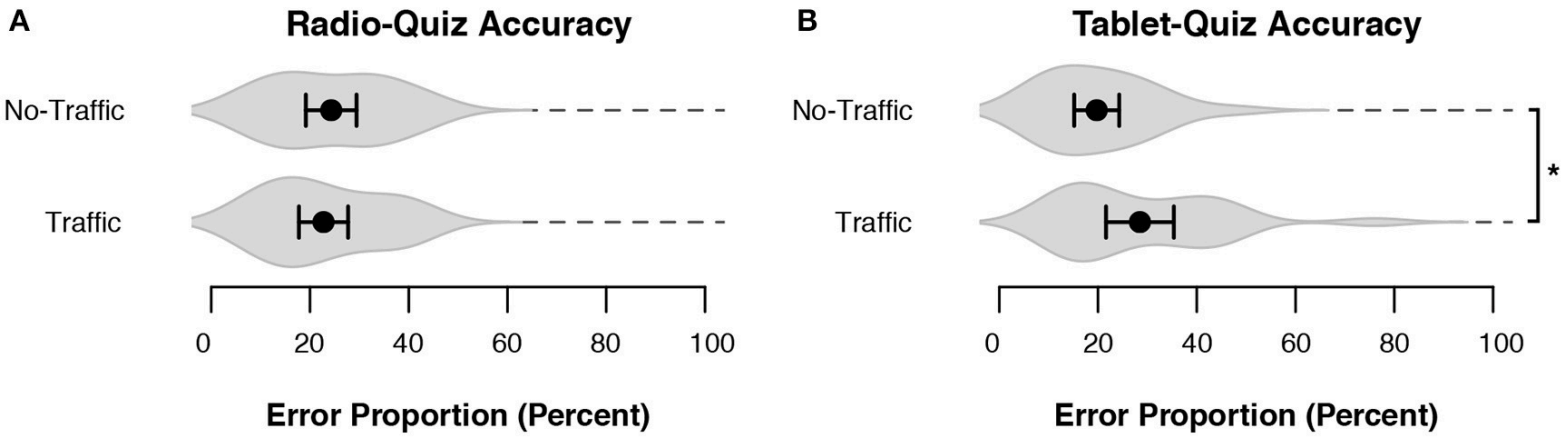
**The performance on the quiz tasks**. Black dots represent the mean across subjects, and bars denote 95% CI. Gray volumes behind the means are (the smoothed estimates of) the underlying distribution of the data (Sheather and Jones, 1991). **(A)** Performance on the Radio Quiz in both driving scenarios. **(B)** Performance on the Tablet Quiz in both driving scenarios. ^*^p < 0.05.

Before examining the driving itself, we will look at the performance on the two quiz tasks. Figure 3A shows that the error proportion of the Radio Quiz was low for both driving scenarios, indicating that participants did perform the secondary task while driving. Figure 3B shows that the error proportion for the Tablet Quiz was similar to the radio quiz. Performance was slightly worse in the Traffic condition than in the No-Traffic condition (*z* = 2.08, β = 0.46, *p* = 0.037).

### Lane deviation and swerving

**Figure 4:**
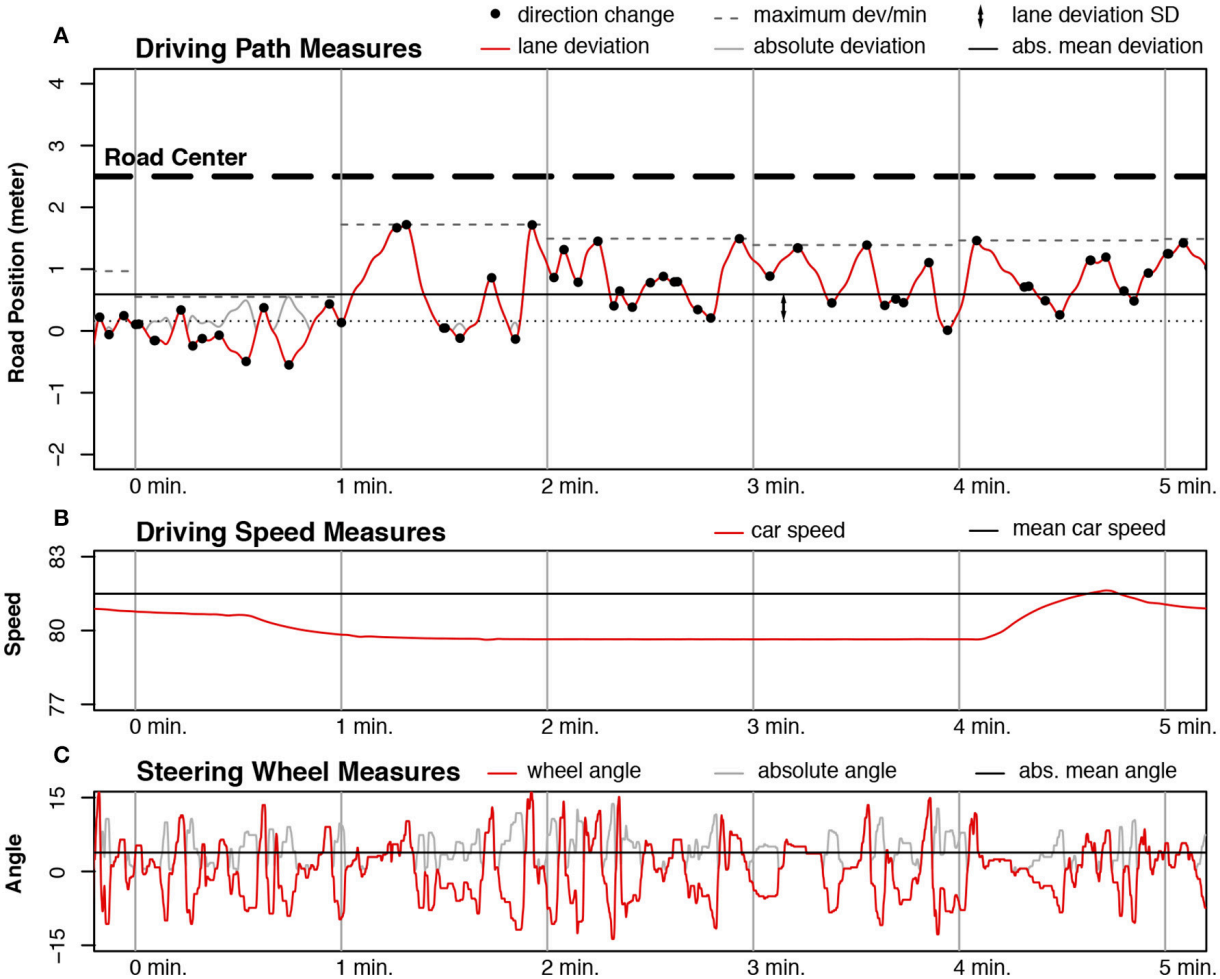
**Measurements taken from a random participant in the No-Traffic condition**. Red lines are the raw data, light gray lines are the absolute values of that data. The segment between two vertical gray lines denotes 1 min of driving. **(A)** Measurements taken from the position of the car. The red line is the deviation from the center of the lane, while the light gray line is the absolute deviation from the lane. The black line with thick dashes is the center of the road, consisting of two lanes. The center of the right lane is denoted by 0 on the y-axis. The solid black line is the average car position. The distance between the solid black line and the dotted black line is the SD of the car position. The black circles located on the dark gray line are direction changes, where the heading of the car shifted from left to right, or vice versa. The dashed gray line segments are the observed maximum absolute deviation from the lane center for each minute of driving time. **(B)** Measurements taken from driving speed. The red line shows the driving speed, while the solid black line is the average observed speed over the block. **(C)** Measurements taken from the steering wheel position. The red line is the steering angle plotted over time, while the light-gray line is the absolute steering angle. The solid black line is the average absolute position of the wheel over the block.

**Figure 5:**
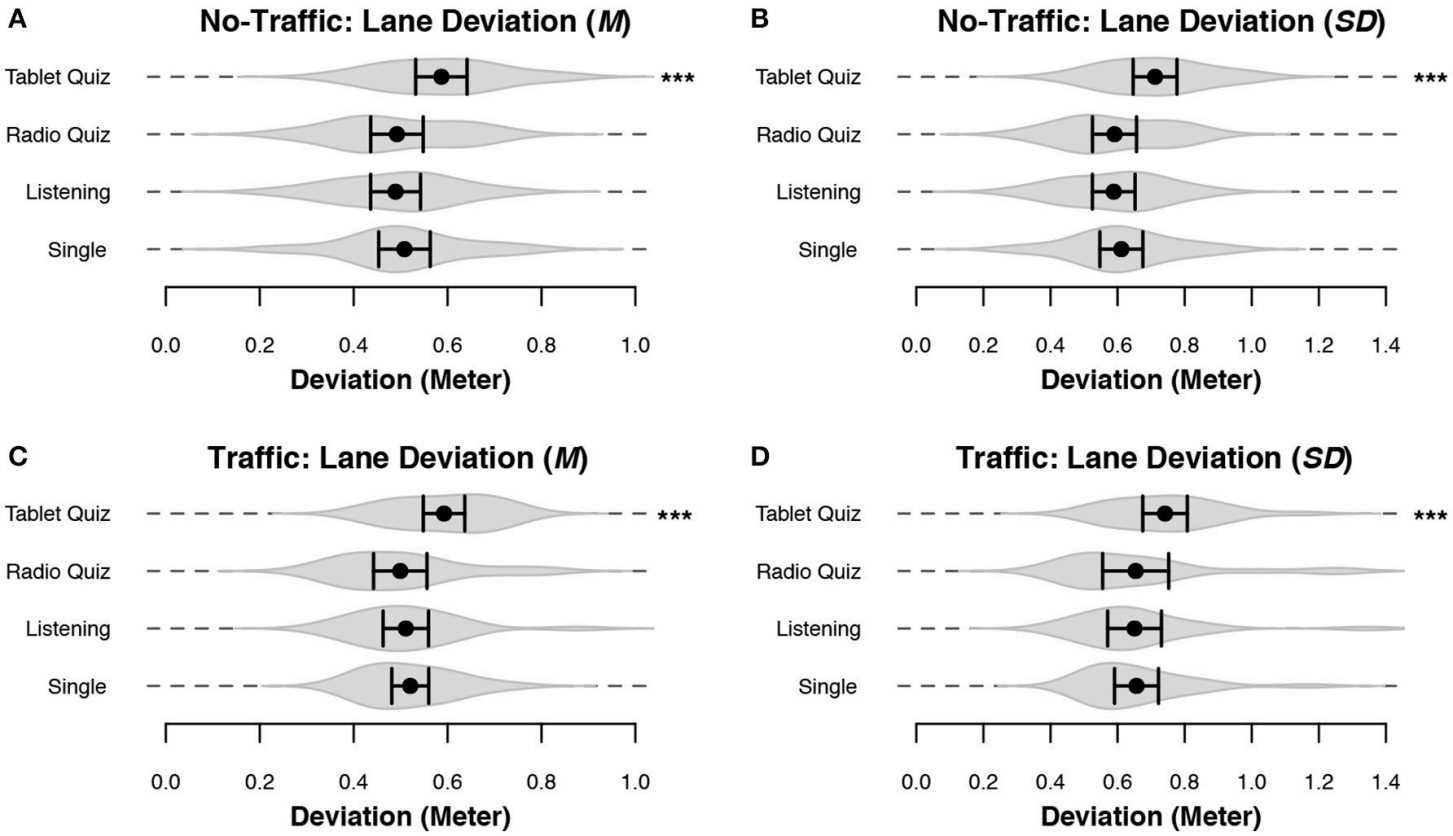
**Lane deviation during the No-Traffic driving scenario and the non-overtaking sections of the Traffic driving scenario**. Black dots represent the mean across subjects, and bars denote 95% CI. Gray volumes behind the means are (the smoothed estimates of) the underlying distribution of the data (Sheather and Jones, 1991). Bars indicate a significant difference between the two indicated conditions. Start without any bars indicate that the condition was significantly different from all other conditions. All data has been demeaned for each participant using the grand mean over all conditions. **(A)** The mean deviation from the center of the right lane in the No-Traffic scenario. **(B)** The standard deviation of the car position in the No-Traffic scenario. **(C)** The mean deviation from the center of the right lane in the Traffic scenario. **(D)** The standard deviation of the car position in the Traffic scenario. ^***^p < 0.001.

The distance to the center of the driving lane, or lane deviation, is a standard measure to investigate driving performance: a large standard deviation indicates a large degree of “swerving” across the road (see Figure 4A). The average and standard deviation of the car position were plotted for both driving scenarios in Figure 5. In all four cases the main effect of condition was significant: the model with condition as fixed effect was better than the model without it for the No-Traffic mean [χ${}_{\left(12\right)}^{2}$ = 38.02, *p* < 0.001], the No-Traffic SD [χ${}_{\left(12\right)}^{2}$ = 38.57, *p* < 0.001], the Traffic mean [χ${}_{\left(12\right)}^{2}$ = 37.58, *p* < 0.001] and the Traffic SD [χ${}_{\left(12\right)}^{2}$ = 25.46, *p* < 0.01]. The values of each participant were demeaned using the grand mean of the participant over all conditions, in order to remove any inherent bias of a participant for a specific position in the lane. For the Traffic scenario all driving segments where participants were overtaking other cars were discarded: These were defined as all data points ranging from 3 s before signaling a lane change to the left lane (using the blinkers) until 3 s after the center of the car crosses the center of the road from the left lane to the right lane, after overtaking a car. The differences between conditions are similar for both driving scenarios: The worst lane-keeping performance occurred when participants had to perform the Tablet Quiz while driving: the degree of swerving was larger, and the average distance to the ideal lane position was also larger. The best performance was obtained when participants were in either the Listening or the Radio-Quiz condition. Consequently, the Single condition results were ranked in the middle of all conditions. However, only the difference between the Tablet-Quiz condition and all other conditions was significant.

**Figure 6:**
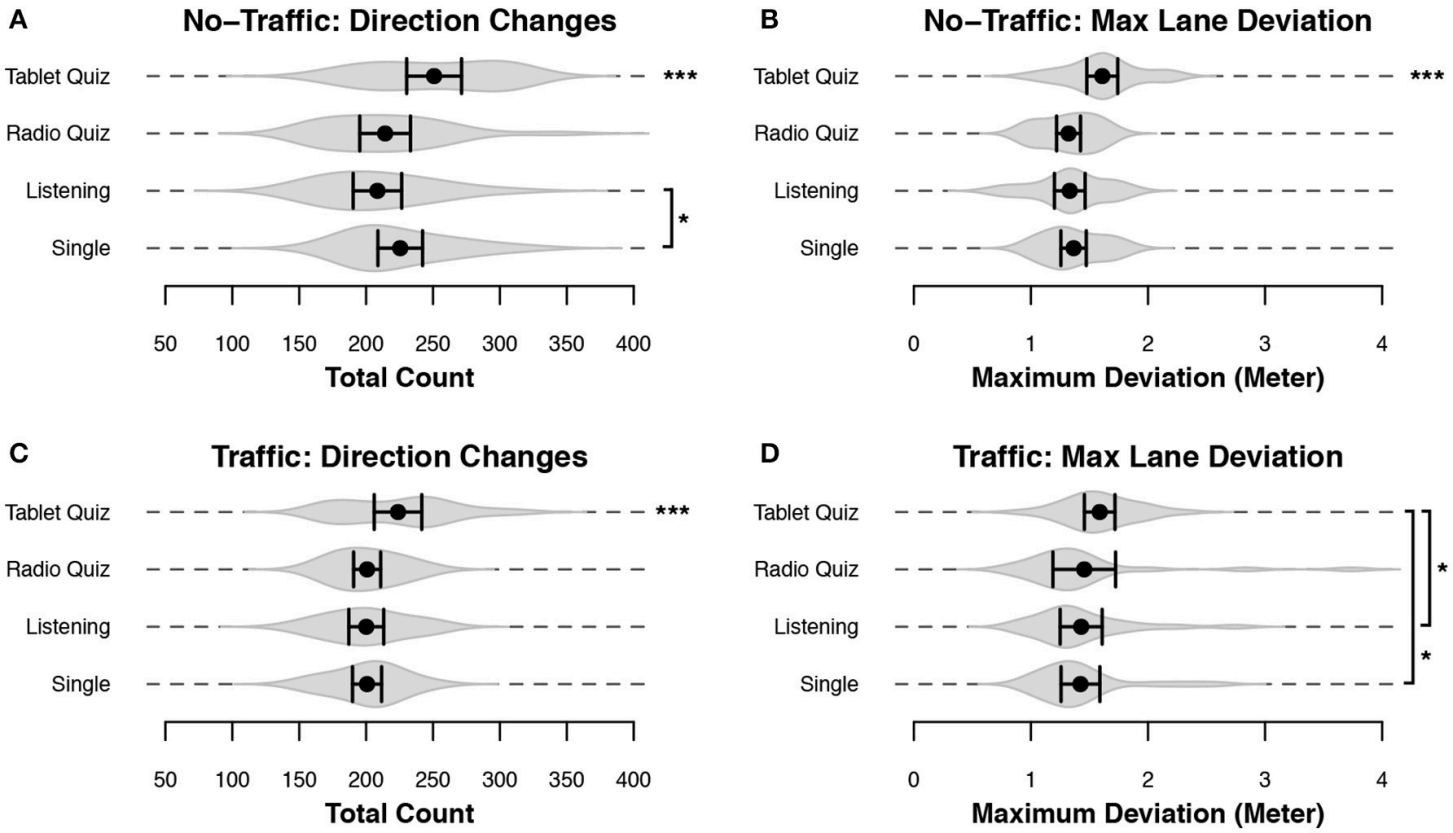
**Performance variables related to lane keeping consistency**. Data for the sparse group were recorded over the entire block, while data of the Traffic group were filtered to remove sections where participants were overtaking other cars. Black dots represent the mean across subjects, and bars denote 95% CI. Gray volumes behind the means are (the smoothed estimates of) the underlying distribution of the data (Sheather and Jones, 1991). Bars indicate a significant difference between the two indicated conditions. Start without any bars indicate that the condition was significantly different from all other conditions. **(A,C)** The number of heading changes made during a block. **(B,D)** The mean of the maximum absolute deviation from the center of the lane, computed for every minute of driving. ^***^p < 0.001, ^*^p < 0.05.

Lane deviation by itself is a limited means of evaluating the consistency and safety of a driver's lane-keeping behavior, as it reduces all the complexities of driving into a single value. In order to study lane keeping in more detail we devised two variables that characterize lane-keeping behavior: the number of changes in car heading, and the maximum observed distance to the ideal lane position (per minute; see Figure 4A). Essentially this divides swerving as calculated by the standard deviation into two separate measures that quantify driving consistency and safety. Both of the measures showed significant differences depending on the secondary task: the model with condition as fixed effect was better than the model without it for the No-Traffic direction changes [χ${}_{\left(12\right)}^{2}$ = 46.99, *p* < 0.001], the No-Traffic maximum lane deviation [χ${}_{\left(12\right)}^{2}$ = 45.36, *p* < 0.001], the Traffic direction changes [χ${}_{\left(12\right)}^{2}$ = 42.85, *p* < 0.001] and the Traffic maximum lane deviation [χ${}_{\left(12\right)}^{2}$ = 34.88, *p* < 0.001]. The directional changes (Figures 6A,C and Table 2) and maximum deviation (Figures 6B,D and Table 2) are consistent with the u-shaped lane deviation results of Figure 5 and Table 1: in both graphs the Tablet Quiz clearly leads to the worst lane-keeping performance, while the Radio-Quiz and Listening conditions tend to result in the best performance. This pattern is most pronounced in the No-Traffic condition. The number of direction changes is higher across conditions in the No-Traffic scenario. This is because there is less data available for the Traffic scenario as the overtake sections have been taken out. Again, the Tablet-Quiz condition is significantly different from all but one of the other conditions. In terms of direction changes the Single condition performs significantly worse than Listening condition in the No-Traffic scenario.

**Table 1:** **Between-conditions comparisons of measurements related to lane deviation in both driving scenarios**.

	**Lane Deviation (*****M*****)**			**Lane Deviation (*****SD*****)**		
	***z***	**β**	***p***	***z***	**β**	***p***
**NO-TRAFFIC SCENARIO**						
Single vs. Listening	1.10	0.019	0.678	1.04	0.023	0.721
Single vs. Radio-Quiz	0.088	0.016	0.809	0.927	0.020	0.785
Radio-Quiz vs. Listening	0.208	0.003	0.999	0.131	0.002	0.999
Tablet-Quiz vs. Single	**3.63**	**0.079**	**<0.01**	**3.94**	**0.101**	**<0.001**
Tablet-Quiz vs. Listening	**4.12**	**0.098**	**<0.001**	**4.55**	**0.123**	**<0.001**
Tablet-Quiz vs. Radio-Quiz	**4.49**	**0.095**	**<0.001**	**4.41**	**0.121**	**<0.001**
**TRAFFIC SCENARIO**						
Single vs. Listening	0.588	0.009	0.556	0.188	0.006	0.851
Single vs. Radio-Quiz	1.18	0.021	0.240	0.085	0.003	0.932
Radio-Quiz vs. Listening	−0.733	−0.012	0.464	0.103	0.003	0.918
Tablet-Quiz vs. Single	**4.01**	**0.072**	**<0.001**	**2.54**	**0.085**	**0.011**
Tablet-Quiz vs. Listening	**4.08**	**0.081**	**<0.001**	**2.46**	**0.091**	**0.014**
Tablet-Quiz vs. Radio-Quiz	**4.25**	**0.093**	**<0.001**	**2.08**	**0.088**	**0.038**

Comparisons were computed by applying a Tukey honest significant difference on the linear mixed-effects models. The resulting z-values, p-values, and estimates (β) are reported. Bold numbers signify significance at the 0.05 level.

**Table 2:** **Between-conditions comparisons of measurements related to lane keeping in both driving scenarios**.

	**Direction Changes**			**Max Lane Deviation**		
	***z***	**β**	***p***	***z***	**β**	***p***
**NO-TRAFFIC SCENARIO**						
Single vs. Listening	**2.38**	**17.04**	**0.018**	0.521	0.033	0.602
Single vs. Radio-Quiz	1.64	11.29	0.101	1.18	0.043	0.239
Radio-Quiz vs. Listening	0.797	5.75	0.426	−0.190	−0.011	0.849
Tablet-Quiz vs. Single	**2.49**	**25.33**	**0.013**	**4.34**	**0.245**	<**0.001**
Tablet-Quiz vs. Listening	**3.86**	**42.38**	<**0.001**	**3.56**	**0.278**	<**0.001**
Tablet-Quiz vs. Radio-Quiz	**5.28**	**36.63**	<**0.001**	**5.89**	**0.288**	<**0.001**
**TRAFFIC SCENARIO**						
Single vs. Listening	0.095	0.583	0.925	−0.080	−0.006	0.936
Single vs. Radio-Quiz	−0.009	−0.042	0.993	−0.424	−0.032	0.672
Radio-Quiz vs. Listening	0.079	0.625	0.937	0.273	0.026	0.785
Tablet-Quiz vs. Single	**3.77**	**23.13**	<**0.001**	**2.24**	**0.164**	**0.025**
Tablet-Quiz vs. Listening	**2.57**	**23.71**	**0.010**	**2.30**	**0.158**	**0.022**
Tablet-Quiz vs. Radio-Quiz	**2.98**	**23.08**	**<0.01**	1.21	0.132	0.225

Comparisons were computed by applying a Tukey honest significant difference on the linear mixed-effects models. The resulting z-values, p-values, and estimates (β) are reported. Bold numbers signify significance at the 0.05 level.

### Steering and speed

**Figure 7:**
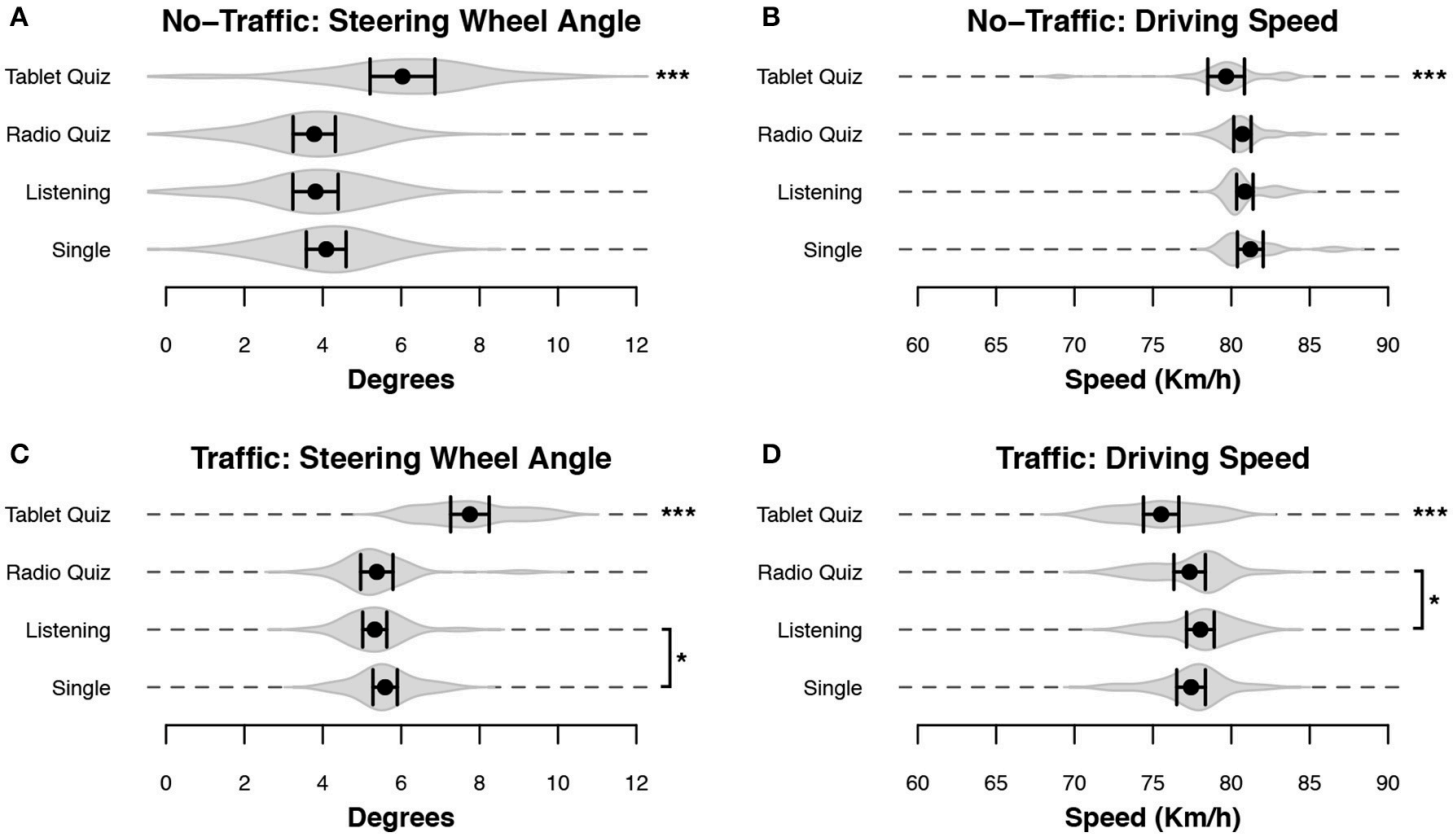
**Driving performance measured for steering and speed variables**. Data for the sparse group were recorded over the entire block, while data of the Traffic group were filtered to remove sections where participants were overtaking other cars. Black dots represent the mean across subjects, and bars denote 95% CI. Gray volumes behind the means are (the smoothed estimates of) the underlying distribution of the data (Sheather and Jones, 1991). Bars indicate a significant difference between the two indicated conditions. Stars without any bars indicate that the condition was significantly different from all other conditions. **(A,C)** The mean absolute angle of the steering wheel position. **(B,D)** The mean driving speed. ^***^p < 0.001, ^*^p < 0.05.

Additionally, driving performance was measured using the steering-wheel and car-speed data (recorded as shown in Figures 4B,C). Both of the measures also showed significant differences depending on the secondary task: the model with condition as fixed effect was better than the model without it for the No-Traffic wheel angle [χ${}_{\left(12\right)}^{2}$ = 91.08, *p* < 0.001], the No-Traffic driving speed [χ${}_{\left(12\right)}^{2}$ = 61.74, *p* < 0.001], the Traffic wheel angle [χ${}_{\left(12\right)}^{2}$ = 150.77, *p* < 0.001] and the Traffic driving speed [χ${}_{\left(12\right)}^{2}$ = 50.35, *p* < 0.001]. Figures 7A,C and Table 3 show that these data are consistent with the lane-keeping data: The significantly larger angle in the Tablet-Quiz condition indicates that participants made sharper steering corrections compared to the other conditions. While steering is related to lane deviation, the steering angle does give different information: the deviation shows the magnitude of swerving across the lane, while the steering angle gives more information regarding how fast corrections were made. Again, the Radio-Quiz and Listening conditions resulted in the best performance, while in the Traffic scenario the Single condition was significantly worse than the Listening condition, but better than the Tablet-Quiz. Across all conditions the steering angle was higher in the Traffic scenario when compared to the No-Traffic scenario. Finally, the average speed shown in Figures 7B,D and Table 3 shows that while participants were able to keep to the instructed speed quite well, the Tablet-Quiz condition consistently led to the slowest driving speed, and was significantly different from all other conditions in both scenarios. In addition, the Radio-Quiz and Listening conditions differed significantly in the Traffic scenario.

**Table 3:** **Between-conditions comparisons of measurements related to driving performance in both driving scenarios**.

	**Wheel Angle**			**Driving Speed**		
	***z***	**β**	***p***	***z***	**β**	***p***
**NO-TRAFFIC SCENARIO**						
Single vs. Listening	1.31	0.273	0.191	1.30	0.347	0.195
Single vs. Radio-Quiz	1.77	0.306	0.077	1.60	0.501	0.110
Radio-Quiz vs. Listening	−0.164	−0.033	0.870	−0.782	−0.155	0.434
Tablet-Quiz vs. Single	**5.53**	**1.95**	<**0.001**	−**2.57**	−**1.54**	**0.010**
Tablet-Quiz vs. Listening	**6.39**	**2.22**	<**0.001**	−**2.33**	−**1.20**	**0.020**
Tablet-Quiz vs. Radio-Quiz	**8.45**	**2.25**	<**0.001**	−**2.17**	−**1.04**	**0.030**
**TRAFFIC SCENARIO**						
Single vs. Listening	**2.17**	**0.265**	**0.030**	−1.31	−0.595	0.190
Single vs. Radio-Quiz	1.35	0.212	0.176	0.230	0.091	0.818
Radio-Quiz vs. Listening	0.443	0.053	0.658	−**2.30**	−**0.686**	**0.021**
Tablet-Quiz vs. Single	**13.8**	**2.16**	<**0.001**	−**4.12**	−**1.91**	<**0.001**
Tablet-Quiz vs. Listening	**14.8**	**2.43**	<**0.001**	−**6.31**	−**2.50**	<**0.001**
Tablet-Quiz vs. Radio-Quiz	**13.1**	**2.38**	<**0.001**	−**5.64**	−**1.82**	<**0.001**

Comparisons were computed by applying a Tukey honest significant difference on the linear mixed-effects models. The resulting z-values, p-values, and estimates (β) are reported. Bold numbers signify significance at the 0.05 level.

With respect to driving speed we see that participants tend to drive slightly slower when they are in the Tablet-Quiz condition, possibly compensating for the extra effort that the secondary task demands. In the No-Traffic scenario the result is that participants in that condition are best in maintaining the proper speed, because driving in the other conditions is too fast. In the Traffic scenario, all driving is slower, even though the result are based on the driving segments without overtaking. As a result, driving in the Table-Quiz condition is worst in this scenario.

### Overtaking

To evaluate how the overtaking of other cars was affected by secondary tasks we considered three variables, the last two of which we developed ourselves for the purpose of this study, given that there are few measures of performance for overtaking in the literature. The first is the number of cars a participant hit, collapsing over hits to left-lane and right-lane traffic. The second variable is accurate turn signal use: Accurate use was defined as using the left turn signal when moving to the left lane and the right turn signal when moving to the right lane. Any other combination, or not using the turn signal at all, was registered as an error. Finally, the overtake-distance was the distance between the participants' car and the leading car at the moment the participants' car crosses the center of the road to overtake that car by switching to the left lane. We are mainly interested in the standard deviation in this distance, with a large variability in overtaking distance indicative of less attentive driving. There is no particular reason to expect a difference in the mean overtaking distance.

Of these variables, all three were significantly affected by condition: the model with condition as fixed effect was better than the model without it for the Cars hit [χ${}_{\left(12\right)}^{2}$ = 23.24, *p* = 0.026], the overtake distance SD [χ${}_{\left(12\right)}^{2}$ = 45.36, *p* < 0.001], and the turn signal use [χ${}_{\left(12\right)}^{2}$ = 137.61, *p* < 0.001]. As we expected, there was no effect of condition on the overtake distance mean [χ${}_{\left(12\right)}^{2}$ = 15.54, *p* = 0.21].

**Figure 8:**
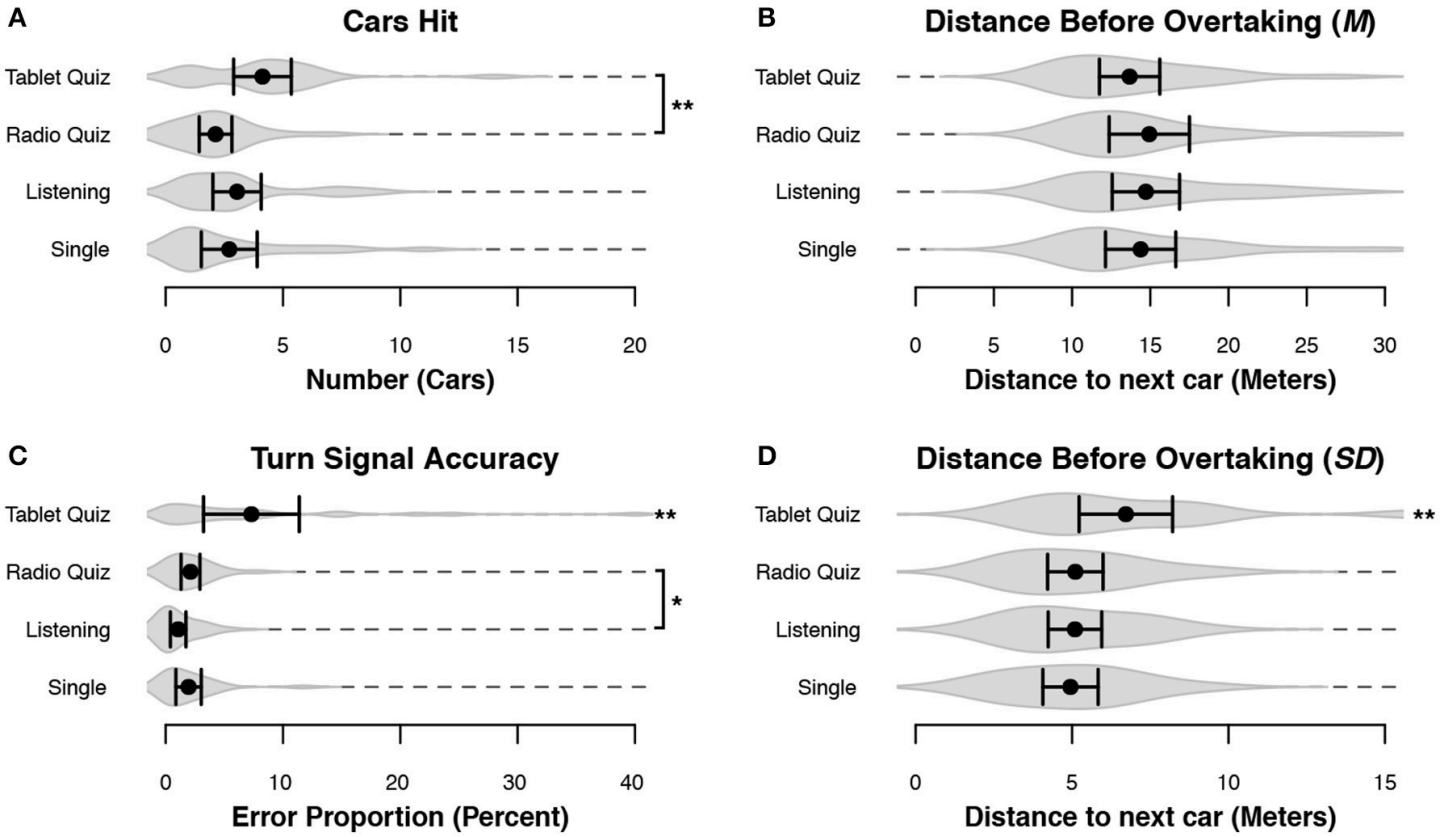
**Measurements of overtaking performance**. Black dots represent the mean across subjects, and bars denote 95% CI. Gray volumes behind the means are (the smoothed estimates of) the underlying distribution of the data (Sheather and Jones, 1991). Bars indicate a significant difference between the two indicated conditions. Start without any bars indicate the condition was significantly different from all other conditions. **(A)** The number of cars the participant connected with. **(B)** The mean distance to the leading car before the participant initiated an overtake maneuver. **(C)** The accuracy of the turn-signal use when changing lanes. **(D)** The standard deviation of the distance to the leading car when initiating an overtake maneuver. ^**^p < 0.01, ^*^p < 0.05.

In accordance with the lane-keeping measurements, all three variables in Figure 8 and Table 4 point toward the Tablet Quiz as the secondary task that resulted in the worst overtaking performance. The Radio Quiz led to the least number of cars hit (Figure 8A), shortly followed by the Single and then Listening conditions. Approximately 88% of the cars that were hit were faster cars in the left lane, while the remaining 12% were slower cars in the right lane: left-lane cars are the cars that will overtake the participant, and hitting them indicates that either the participant did not see that car, or misjudged when that car would overtake the participant. The right-lane cars are the cars the participant had to overtake. Hitting them indicates that participant did not steer accurately (while overtaking), or misjudged the speed at which the other car was moving, as this speed varied over time. In terms of turn-signal use (Figure 8C) the Listening condition outperformed all others, with the Radio-Quiz and Single conditions sitting in the middle. Figures 8B,D present the differences in overtake distance. The only condition that stands out is the Tablet Quiz: performance of the other conditions was similar for both the average and the standard deviation.

**Table 4:** **Between-conditions comparisons of measurements related to overtaking actions**.

	**Cars Hit**			**Overtake Distance (*****M*****)**		
	***z***	**β**	***p***	***z***	**β**	***p***
Single vs. Listening	−0.578	−0.333	0.563	0.420	0.335	0.675
Single vs. Radio-Quiz	1.00	0.583	0.317	0.811	0.554	0.417
Radio-Quiz vs. Listening	−1.43	−0.875	0.153	−0.256	−0.219	0.798
Tablet-Quiz vs. Single	1.62	1.42	0.104	1.07	0.704	0.284
Tablet-Quiz vs. Listening	1.41	1.08	0.160	1.82	1.04	0.068
Tablet-Quiz vs. Radio-Quiz	**3.00**	**2.00**	**<0.01**	1.85	1.26	0.064
	**Turn Signal**			**Overtake Distance (*****SD*****)**		
	***z***	**β**	***p***	***z***	**β**	***p***
Single vs. Listening	−1.48	−0.826	0.139	−0.407	−0.144	0.684
Single vs. Radio-Quiz	0.816	0.292	0.414	−0.482	−0.155	0.630
Radio-Quiz vs. Listening	−**2.27**	−**1.12**	**0.023**	0.033	0.011	0.974
Tablet-Quiz vs. Single	−**2.58**	−**0.997**	**<0.01**	**2.85**	**1.77**	**<0.01**
Tablet-Quiz vs. Listening	−**3.37**	−**1.82**	**<0.001**	**2.98**	**1.63**	**<0.01**
Tablet-Quiz vs. Radio-Quiz	−**2.25**	−**0.704**	**<0.025**	**2.61**	**1.61**	**<0.01**

Comparisons were computed by applying a Tukey honest significant difference on the linear mixed-effects models. The resulting z-values, p-values, and estimates (β) are reported. Bold numbers signify significance at the 0.05 level.

The act of overtaking might reduce secondary task performance if the driving task is prioritized. For both the Radio-Quiz and Tablet-Quiz tasks we investigate how likely drivers would answer questions during overtaking when that question was prompted during the first half of an overtake maneuver. If the secondary tasks were not affected by overtaking, we would expect to find a ratio around 1:1 between responding during overtaking and responding afterwards. However, we found that drivers were more likely to respond to questions after the overtake maneuver was completed for both the Radio Quiz (65%; *p* < 0.001 given P(H) = 50%) and Tablet Quiz (68%; *p* < 0.001 given P(H) = 50%). When examining the accuracy on questions answered during overtaking we found a reduction in accuracy for the Tablet Quiz. However, neither the difference in the Tablet Quiz (73 vs. 54%; β = −0.619, *z* = −1.58, *p* = 0.11) nor the difference for the Radio Quiz (77 vs. 76%; β = 0.119, *z* = 0.378, *p* = 0.705) reached significance.

### Summary of empirical results

When taken together, the measurements we computed using the driving data present a comprehensive evaluation of driving consistency and safety under varying different loads for working memory, perception, and motor actions. The influence of secondary tasks on driving performance was compared by ranking the performance given each secondary task per variable for each of the scenarios as presented in Table 5: ranks were assigned from worst performance (−) to best performance (++) according to the averages presented in earlier plots. For an analysis of the ranked data we refer to the Appendix in Supplementary Material.

**Table 5:** **A ranking of all measurements made for the No-Traffic and Traffic scenarios, as well as the measurements related to overtaking**.

	**Single**	**Listening**	**Radio Quiz**	**Tablet Quiz**
**NO-TRAFFIC**				
Lane Deviation (*M*)	−	++	+	–
Lane Deviation (*SD*)	−	++	+	–
Direction Changes	−	++	+	–
Max Lane Deviation	−	+	++	–
Steering Wheel Angle	−	+	++	–
Driving Speed	–	−	+	++
**TRAFFIC**				
Lane Deviation (*M*)	−	+	++	–
Lane Deviation (*SD*)	−	++	+	–
Direction Changes	+	++	−	–
Max Lane Deviation	++	+	−	–
Steering Wheel Angle	−	++	+	–
Driving Speed	+	++	−	–
Car Hits	+	−	++	–
Overtake Distance (*M*)	−	+	++	–
Overtake Distance (*SD*)	++	+	−	–
Turn Signal Accuracy	−	++	+	–

*The ranking of best performance to worst performance is:* ++, +, −, *and finally –.*

The Tablet Quiz is the worst scoring secondary task, ranking lowest in almost all variables across both scenarios. The remaining three conditions see some variation across scenarios: In the No-Traffic scenario, the Single condition resulted in the lowest performance after the Tablet-Quiz condition, while the Radio-Quiz and Listening conditions scored equally well. In the Traffic scenario the Single condition remains the second-lowest performing. However, there is a difference between Radio Quiz and Listening, with the Radio Quiz leading to slightly lower driving performance overall. Looking at the variables related to overtaking, the order of the conditions is much less defined (with the exception of the Tablet Quiz): The Single condition leads to performance that is only slightly worse than the two aural conditions. The two remaining conditions, Listening and Radio Quiz, showed performance similar to each other.

## Discussion

In this study we expanded on previous research regarding the positive and negative effects of multitasking during driving: We compared different secondary tasks and driving scenarios within a single paradigm. Furthermore, we used typical commute durations of 30 min for each condition to approximate realistic driving circumstances. This paradigm allowed us to compare secondary tasks and driving scenarios based on the cognitive requirements placed on the driver. To summarize, we found that when ordering the different secondary task conditions based on the expected interference with driving (i.e., No secondary task, Listening, Radio-Quiz, and Tablet-Quiz), a u-shaped pattern appears that was consistent across most of the measurements. This pattern indicated that the Tablet-Quiz that had the largest resource overlap with driving resulted in the worst driving performance, while the Listening and Radio-Quiz led to the best driving performance—better than not having a secondary task. The caveat is that the difference between the No secondary task and the two Radio conditions is often not significant in the individual comparisons, even though a combined rankings analysis (see Appendix in Supplementary Material) gives use a significant result. But given the non-standard nature of that analysis we have to conclude that the difference is tentative. The overtaking data did not show a consistent pattern, except for the visual tablet task, which led to significantly lower driving performance across measurements.

Thus, all results clearly show that the Tablet-Quiz, a visual secondary task, leads to the worst driving performance across measures. However, the result that stands out was that driving without a secondary task did not lead to the best performance; instead listening to the radio might be slighly better. This is in line with earlier research on monotonous driving conditions with very sparse traffic (Gershon et al., 2009; Atchley and Chan, 2010). We extended these results to a driving scenario with a substantial number of vehicles. Under these circumstances the driving task is more engaging because the driver must monitor, and react to, traffic.

These results fit the expectations we stated in the introduction with respect to the No-Traffic condition. However, we expected the working memory load of the Traffic condition to be sufficient to lead to best performance in the Single task condition, whereas the results show a pattern that is similar to the No-Traffic condition. Possibly the working-memory load in the Traffic condition was too modest.

As discussed in the introduction, at least two theories can explain why driving without a secondary task might lead to worse performance under both circumstances. Execution-focus theories imply that increasing the step-by-step attentional control of skilled processes—which is the case with driving as a single task—might disrupt proceduralized processes in sensorimotor tasks (Baumeister, 1984; Beilock and Carr, 2001). Actions that are normally executed as a single uninterrupted unit are divided up it to smaller units that are executed separately, leading to slower actions. Thus, over-regularization of the driving task can lead to a decrease in performance because the actions that must be performed are slowed down. This is similar to explicitly thinking of how each foot is placed while walking. The issue with these theories is that they assume that the driver is under significant performance pressure, which is unlikely in our paradigm for both traffic scenarios.

Another possibility is that while the traffic scenario that we used is not quite as monotonous as the sparse-traffic scenarios in previous studies, it is still quite a repetitive sequence of lane keeping and overtaking, which likely leads to boredom over a 30-min drive. This might cause people to shift focus toward internal processes, resulting in a decoupling from the external environment (Cheyne et al., 2009; Smallwood and Schooler, 2015). This type of mind wandering can have a significant negative impact on driving behavior because it affects how well a driver observes the surroundings (He et al., 2011). In terms of the threaded cognition framework, mind wandering and other forms of distraction can be explained by the assumption that we are always looking for things to do with unused mental resources (Katidioti and Taatgen, 2014). Mind wandering probably needs declarative memory and working memory, but may extend its needs to other resources. As long as the resources that are required for driving are not claimed by mind wandering, driving performance is not affected. However, mind wandering may lead to mental activities that are in conflict with driving, for example if mental imagery is part of the train of thoughts. In one of our experiments, subjects performed poorer on a complex working memory task if it involved words that prompt mind wandering. This decrement in performance could be explained by a model in which mind wandering used resources that were needed for rehearsal (Daamen et al., 2016). Given the lack of performance pressure in the single-task condition of the experiment, this seems the more likely explanation. To further test this possibility, it would be interesting to measure mind wandering during driving directly, for example with EEG or pupil dilation (e.g., Mittner et al., 2014).

In general, the effects we found were small, with differences typically in the order of 10%. People seem to adapt their behavior well to the driving circumstances. This is most evident in the average driving speed presented in Figures 7B,D: The difficulty of the visual tablet task leads drivers to slow down. In addition, there are indications that drivers prioritize the driving task during overtaking, given that they are inclined to postpone a response to a task until the maneuver has been completed. Despite this, concurrent performance of the task involving a tablet did clearly lead to the worst overtaking performance. This result could be explained if we assume that even during overtaking an occasional switch of attention to the secondary task would occur: a glance at the tablet is more costly compared to the other tasks, as visual interference has a larger impact on driving performance than aural interference because the environment can no longer be monitored.

The results of this study provide a more complete understanding regarding the interaction between secondary tasks and driving circumstances and the resulting driving performance. Essentially, the observations are in line with current theories of multitasking (Wickens, 2002; Salvucci and Taatgen, 2008): performance is primarily reduced when there is a resource conflict. A driving scenario with no right-lane traffic has low working-memory and motor load. Under these circumstances the driving task is complemented by tasks that require aural and working-memory facilities, as there is no resource overlap. While driving without a secondary task is expected to lead to the highest driving performance, it is also the condition with the highest risk of mind wandering. The effects of mind wandering are consistent with the observed driving performance: mind wandering can lead to a narrowed visual focus, which could cause insufficient monitoring of the environment (He et al., 2011). Finally, a visual task interferes strongly with the driving task even when there is no traffic to account for, as lane keeping still requires constant visual attention.

When there is other traffic that needs to be reacted to, driving has a much higher working-memory and motor load: the location of surrounding vehicles has to be monitored, and the environment has to be navigated (Gugerty, 1997). As a consequence, driving performance was affected when a secondary task required additional information needed to be maintained in working memory: a concurrently performed aural working-memory task led to worse driving performance than an aural listening task, primarily seen in the average driving speed. This is not observed when there is no surrounding traffic, thus it is likely due to the overlap in working-memory requirements. The effect is only minor, however, as driving without a secondary task still leads to comparatively worse driving performance. A visual working-memory task again leads to the lowest driving performance, as there is overlap in two crucial resources.

The study we performed shares similarities with the work by Gershon et al. (2009) and Atchley and Chan (2010), who both showed that a secondary task during driving could have a positive effect. However, whereas these previous investigations tested the effect of a single secondary task on driving performance in a single driving scenario, we tested a range of different tasks with different resource requirements, under different driving conditions. The paradigm we used made it possible to directly compare secondary tasks, as well as investigate the interaction between secondary task and driving circumstances. This allowed us to determine that a simple listening task complements driving more consistently under varying traffic circumstances than the relatively complex tasks used by Gershon et al. (2009) and Atchley and Chan (2010). Furthermore, we found no evidence that a more involved secondary task can have a stronger positive effect on driving than the passive listening task.

To conclude, safe multitasking during driving depends on engaging in tasks that complement the requirements of driving at that particular time. When the driver is fully engaged, such as driving through city traffic, it is best to focus fully on driving as indicated by previous research (Stein et al., 1987; Neyens and Boyle, 2007). However, on roads with low traffic density the driving task is much less demanding, and may lead to mind wandering. Such an internally focused distraction can lead to bad driving performance because the environment is no longer monitored sufficiently. While mind wandering is not as dangerous as a visual distraction during driving, this work shows that it might be sensible to engage in mildly distracting activities such as listening to the radio. These can prove beneficial to driving performance by providing a less interfering task alternative.

## Author contributions

MN: Designed and created experiment, ran experiment, wrote first version manuscript. JB, NT, and HR: Helped design experiment, reviewed manuscript, wrote revisions.

### Conflict of interest statement

The authors declare that the research was conducted in the absence of any commercial or financial relationships that could be construed as a potential conflict of interest.

## Footnotes

^1^According to Jeffreys (1961, p. 432), a Bayes Factor <1/3 qualifies as substantial evidence against the alternative hypothesis (in this case that the groups are different).
