# Supplementary material for: Driving and Multitasking: The Good, the Bad, and the Dangerous
Source: Front Psychol. 2016 Nov 8;7:1718. doi: 10.3389/fpsyg.2016.01718 (PMC5100650; doi:10.3389/fpsyg.2016.01718)
Supplement: Supplementary file 1 [file DataSheet1.docx]

# Appendix: Integrated Driving Performance

Here we report the performance differences between conditions and driving scenarios when integrating the different measurements. When combined, the measurements we computed using the driving data present a comprehensive evaluation of driving consistency and safety under varying cognitive and peripheral loads. For each dependent variable an order can be determined per participant that ranks the four different conditions from worst to best performance. From best to worst, conditions were given a rank from 0 to 3 depending on the performance of the particular participant. To visualize these rankings they were summed to determine a total score per condition, the averages of these scores can be seen in Figure 1. A higher score indicates worse overall performance for that condition. To score the lane keeping performance we summed the ranks of average lane deviation, directional changes, max deviation, wheel angle, and average speed. The limitation of this scoring approach is that it is based on an ordinal scale, and therefore not continuous. However, it is the best method we have available to integrate the dissimilar measurements of driving performance.

Figure 1. Aggregated performance scores for each condition. Black dots represent the mean across subjects, and bars denote 95% CI. Gray volumes behind the means are (the smoothed estimates of) the underlying distribution of the data (Sheather & Jones, 1991). *Panel A*: The mean penalty for driving performance during the No-Traffic scenario. *Panel B*: The mean penalty for driving performance during the non-overtaking sections of the Traffic scenario.

To determine if the ranks achieved by participants were significantly different between conditions, we computed cumulative link models (Agresti, 2002) for both traffic scenarios. The output of these models can be seen in Table 1. Integrated driving performance scores for the No-Traffic (Figure 9A) and Traffic (Figure 9B) scenarios followed the same u-shaped pattern seen in plots of individual measurements in the main text. The Tablet-Quiz has the highest average aggregated score, which was significantly different from the remaining three conditions. The scores of the Listening and Radio-Quiz conditions were significantly lower than the Single condition in both driving scenarios, and thus the two auditory conditions led to the best performance on average.

Table 1. Between-conditions comparisons of ranked measurements related to lane keeping in both driving scenarios. The resulting *z* values, *p* values, and estimates (*β*) are reported. The *p*-values were corrected for multiple comparisons using a False Discovery Rate correction. Bold numbers signify significance at the 0.05 level.

|  | No-Traffic | | |  | Traffic | | |
| --- | --- | --- | --- | --- | --- | --- | --- |
|  | *z* | *β* | *p* |  | *z* | *β* | *p* |
| Single vs. Listening | **-2.61** | **-.562** | **.014** |  | **-2.21** | **-.463** | **.033** |
| Single vs. Radio-Quiz | **-2.27** | **-.484** | **.028** |  | **-2.47** | **-.524** | **.020** |
| Radio-Quiz vs. Listening | -.371 | -.078 | .711 |  | .289 | .061 | .773 |
| Tablet-Quiz vs. Single | **-8.89** | **-2.19** | **< .001** |  | **-9.48** | **-2.40** | **< .001** |
| Tablet-Quiz vs. Listening | **-11.0** | **-2.75** | **< .001** |  | **-11.1** | **-2.87** | **< .001** |
| Tablet-Quiz vs. Radio-Quiz | **-10.8** | **-2.67** | **< .001** |  | **-11.3** | **-2.93** | **< .001** |

# References

Agresti, A. (2002). *Categorical data analysis* (2nd ed.). Wiley.

Sheather, S. J., & Jones, M. C. (1991). A reliable data-based bandwidth selection method for kernel density estimation. *Journal of the Royal Statistical Society (B)*, *53*, 683–690.
